# The influence of task constraints on football players’ performance: non-linear trends during goalkeeper training

**DOI:** 10.5114/biolsport.2026.163368

**Published:** 2026-07-13

**Authors:** Elliot Bastias, Fabian W. Otte, James Vaughan, Dennis Hörtin, Mark O’Sullivan

**Affiliations:** 1 Research and Development Department, AIK FF, Stockholm, Sweden; 2 Independent Researcher, no affiliation with organization, United Kingdom; 3 Te Huataki Waiora – School of Sport and Human Movement, University of Waikato, Hamilton, New Zealand; 4 National Research Center for Youth Sports, Karlstad University, Karlstad, Sweden; 5 Department of Sport and Social Sciences, Norwegian School of Sport Sciences, Oslo, Norway

**Keywords:** Sport complexity and performance, Constraints-Led Approach (CLA), Non-Linear Pedagogy (NLP), Individualized athlete development, Athlete-environment couplings

## Abstract

In the realm of sports, task constraints shape players’ adaptation processes and serve as crucial regulators of performance. While previous studies have examined the influence of task constraints on performance using simple linear models, potential non-linear relationships remain poorly understood, especially in highly complex training contexts such as football goalkeeping. Our study involved monitoring the performance coefficients of six female youth goalkeepers across 22 training tasks, which were characterized by eight task constraints encompassing interactions among players and properties of the training environment. We used Generalized Additive Mixed Models (GAMMs) to examine potential non-linear relationships between task constraints and goalkeeper performance. GAMMs revealed significant non-linear effects for several task constraints, i.e., *the number of players, field dimensions, spatial degrees of freedom and playing time (edf* = 2.1–3.3, *P* < 0.001), explaining 20.8–30% of deviance. Landscape representativity showed a significant overall effect on performance (*P* = 0.003). The results indicated that task constraints may influence goalkeepers’ performance with potential threshold-like effects and, thus, the manipulation of task constraints during training might be partially context-dependent. Overall, the results advocated for a constraint-led approach that facilitates representative training designs high in player interactions to devise impactful training environments for players’ skill development.

## INTRODUCTION

The development of skill and performance in sport is a complex interplay between players and their surrounding environments [1, 2, 3]. Players adapt to their own and others’ perceptual and physical abilities, the specific task at hand, and the environmental conditions [4, 5, 6]. As such, the players, the task, and the environment are essential constraints shaping processes of adaptation, coordination, and skill acquisition over varying time scales [7, 8, 9, 10]. While qualitative studies have investigated how constraints may influence performance [11, 12, 13], there is a need for quantitative research to further elucidate the influence of constraints on performance, particularly including non-linear models that may better capture the complex nature of sport.

### The role of task constraints and non-linearity on players’ performance

In quantitative terms, the effect of task constraints on players’ performance has been previously studied. Task constraints such as the number of players, team imbalances, and training area size can significantly influence the physiological, technical, and tactical behaviors of players [14, 15, 16, 17]. Performance is also influenced by training frequency [18, 19]. Here, performance outcomes typically progress rapidly at the beginning and then slow down over time [20, 21]. Yet, previous research has mostly used simple linear models to explore the relationships between task constraints and performance, ignoring how complex (i.e., non-linear) these relationships could be [e.g., 22].

In fact, modern movement theories suggest that players and teams operate as *complex adaptive systems* [23, 24, 25, 26]. This indicates that an increasing number of constraints in a model enhances insights into performance outcomes [22, 27, 28, 29]. However, integrating diverse constraints into performance analysis can be challenging due to complex interactions among variables [27, 28]. While the investigation of univariate relationships between task constraints and performance outputs may simplify the statistical framework and enhance feasibility, it is imperative that univariate models retain the flexibility necessary to accommodate non-linear trends. In response to this challenge, generalized additive models (GAMs) have been employed to explore the degree of non-linearity inherent in complex univariate relationships across diverse fields, including ecology, psychology, and sociology [e.g.,^30^]. These models effectively incorporate both parametric functions (i.e., linear terms) and non-linear functions, allowing for an estimation of the “wiggliness” (i.e., degree of non-linearity) of the relationships under investigation. Therefore, GAMs present an appropriate tool for the flexible evaluation of nonlinear relationships [31]. Despite their demonstrated utility, the application of GAMs to assess the influence of task constraints on performance within sports—particularly regarding potential non-linear trends—remains insufficiently explored.

### Non-linear dynamics in team sports: a study case of football goalkeeping

The exploration of non-linear relationships between task constraints and performance might be especially relevant in highly complex sports contexts such as football goalkeeping [22, 32, 33]. This question is critical since football goalkeepers (GKs) have a major influence on their team’s goal-preventing as well as attacking moments [32, 34, 35] over changing game dynamics [2, 36]. Given this high complexity in goalkeeping interactions, research designs that are player-task-environment driven, such as “Periodization of Skill Training” (PoST), have been reported to enrich our understanding of skill acquisition and performance in goalkeeping [37, 38]. Based on this framework, a quantitative study on professional GKs has found that task constraints may explain 47% of GKs’ performance [22]. This study identifies numerical complexity (i.e., the number of potential interactions among players within the task) to be an important factor regulating performance, and this factor seems to influence performance following nonlinear (i.e., asymptotic) trends. However, this study is limited by a small sample size, and further quantitative research is needed to fully understand the complex relationships between task constraints and GKs’ performance.

The purpose of this study is to investigate the relationships between task constraints and GKs’ performance, with a particular focus on the degree of non-linearity within these relationships. To do that, we monitored GKs’ performance across 22 training tasks, including six GKs (belonging to U16–U17 age groups). Study tasks were distinguished by eight task constraints encompassing interactions among players and properties of the training environment (see Figure 1 and methods). For each training task and GK, we estimated a performance coefficient (PC) based on goal-oriented actions that assessed each GK’s effectiveness (Figure 1). To explore the degree of non-linearity of these relationships, we used GAMs, which included parametric and non-parametric estimations (see statistical methods). We hypothesized that task constraints would explain GKs’ performance, as these constraints influence the spatial and temporal informational boundaries for GKs to achieve competitive aims. We also hypothesized that relationships between task constraints and performance would show some degree of non-linearity, reflecting the complex nature of goalkeeping. Overall, this study provides preliminary quantitative evidence on how task constraints may influence GKs’ performance within the specific training context investigated.

**FIG. 1:**
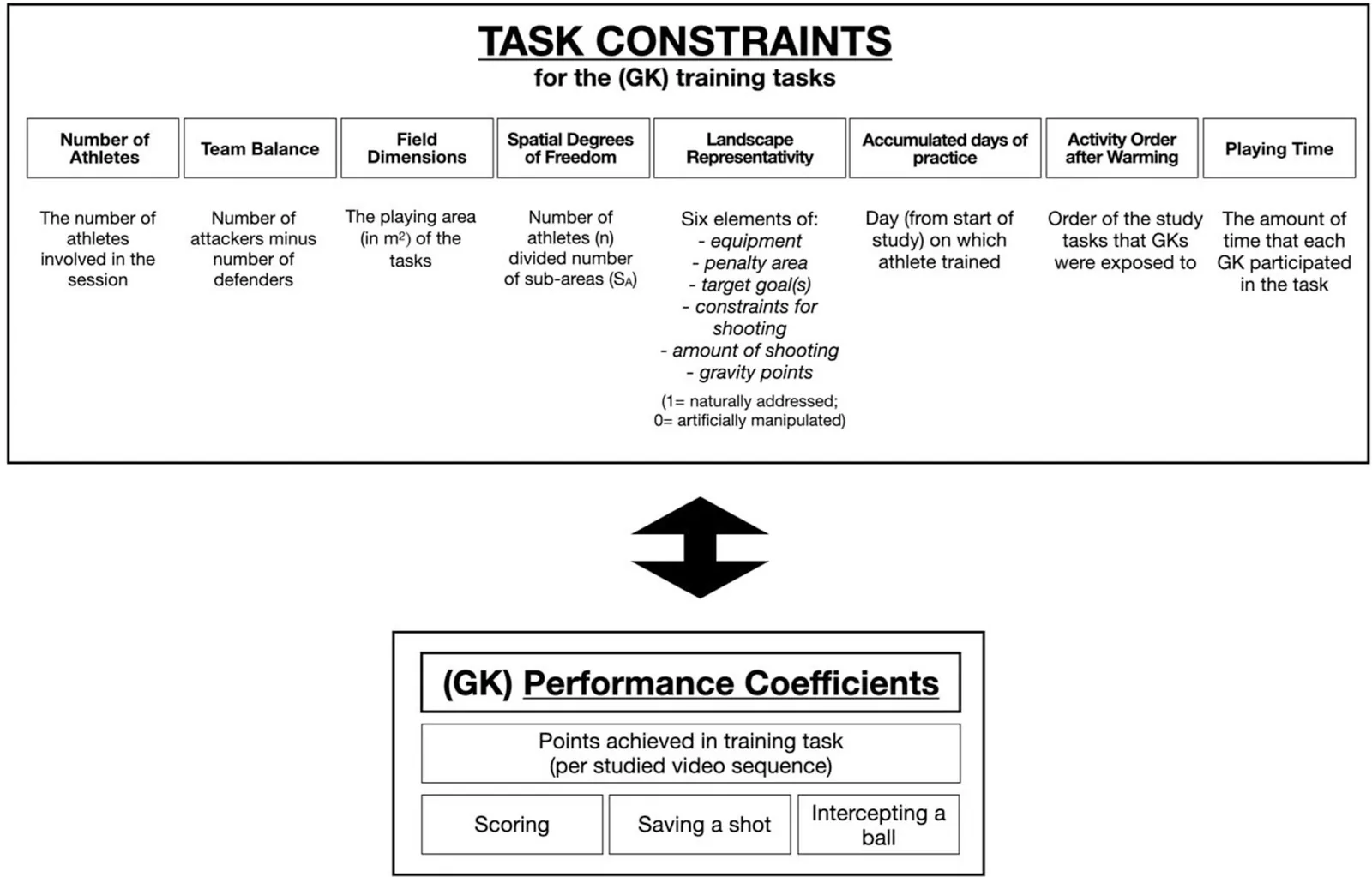
Overview of the eight study constraints and performance Coefficients (PC) for the studied football GK training tasks.

## MATERIALS AND METHODS

### Participants

In this study, six elite female GKs (GK1 to GK6) from two age-group squads (U16: GK1, GK2, GK3; U17: GK4, GK5, GK6) were selected for analysis. Both squads competed at the highest level of the Swedish youth national football league. To simulate “match-like” conditions, some tasks included outfield players. The coaching team included a head of goalkeeping with a UEFA Goalkeeping B diploma and two additional GK coaches, alongside a head coach, two assistant coaches, and a physical performance coach, all holding UEFA B diplomas. Informed consent was obtained from participants and their parents for video recordings. The video analysis was conducted retrospectively, guaranteeing that the intervention remained non-invasive for the GKs involved. The data analyzed in this study were retrospectively obtained from routine performance monitoring conducted as part of the club’s standard practice. As the dataset was anonymized prior to analysis and no intervention was applied, formal ethical approval was not required under applicable regulations. Performance actions were coded independently by the three trained goalkeeping coaches following predefined scoring criteria. Inter-rater reliability was assessed using Fleiss’ kappa, yielding a value of κ = 0.98, indicating a very high level of agreement among the three evaluators.

### Procedure

#### Training tasks

The present study was conducted over a duration of 136 days, initiating on January 17^th^ and concluding on May 23^rd^, 2023. This period encompassed both the preseason phase and the initial segment of the competitive league season. Throughout this timeframe, GKs participated in two distinct categories of training tasks: GK-specific tasks (n = 11), in which only GKs and the GK coaches were involved, and team-oriented tasks comprising small-sided games and larger-scale scenarios (n = 11), facilitating interaction between GKs and outfield players, in accordance with the methodologies established by Bastias et al. [22]. Team-oriented tasks were implemented separately with the U16 and U17 squads, yielding 22 tasks (i.e., 11 for U16 and 11 for U17). One task from each age group was excluded because performance coefficients could not be calculated according to the minimum action criterion (see Performance coefficients of the GKs section). Consequently, 20 team-oriented tasks were retained for analysis. Therefore, the study included a total of 31 tasks (11 GK-specific, 10 from U16, and 10 from U17). The training tasks were meticulously designed to be ecologically valid, adhering to the task design framework elaborated by O’Sullivan and colleagues [39]. Furthermore, each training session commenced with a structured physical warm-up routine, derived from the FIFA11+S program [40], aimed at preparing both the upper and lower extremities while simultaneously reducing the risk of injuries among GKs.

#### Task constraints

The design of the training tasks was informed by eight task constraints, as delineated in Bastias et al. [22]. These constraints encompassed factors such as: (1) the *number of players*, (2) *team balance* (i.e., the number of attackers minus the number of defenders), (3) *field dimensions*, (4) *spatial degrees of freedom* (i.e., the quotient between the number of players and the number of training sub-areas for players to move), (5) *landscape representativity* (based on six major landscape elements within the task), (6) *accumulated days of practice* (indicating the number of total days of training over the study period, as training recurrence), (7) the *activity order after warming* (to include the effect of the order of tasks after GKs warm-ups), and (8) *playing time* of the training task for each GK, as training duration (see Bastias et al. [22] for further details).

### Performance coefficients of the GKs

The performance coefficients (PC) were utilized to evaluate the effectiveness of each GK based on specific actions performed during a task, such as scoring a goal, saving the opponent’s shot on target, or intercepting and distributing the ball to a specified area [22]. To ensure accuracy and consistency, points for these actions were retrospectively allocated by analyzing video recordings of each task. The total points earned by each GK were calculated alongside the total potential points based on the number of attempts. For example, in a shooting task, GKs received one point for saving a shot, and the total points were divided by the total number of shots on target. The PCs for each task and GK were then calculated by dividing the number of points achieved by the number of attempts, resulting in a quotient from 0 to 1 [22]. Tasks with less than five actions for a GK were excluded from the calculation of PC, as a minimum of five actions (N = 5) was assumed.

### Statistical analysis

#### Sample size

Each GK participated in a maximum of 22 training tasks (i.e., 11 GK-specific and 11 team-oriented tasks). Across the six GKs (N_GKs_ = 6), this yielded a potential maximum of 132 observations. After excluding observations that did not meet the minimum action criterion for PC estimation and missing values, the final dataset included 102 valid observations. Given the applied elite training context and the limited number of available GKs, this study should be interpreted as an exploratory case-series investigation rather than a design intended for broad statistical generalization.

#### Generalized Additive Models (GAMs)

We used Univariate Generalized Additive Mixed Models (GAMMs) to examine potentially non-linear relationships between task constraints and GKs’ performance. Continuous predictors (*number of players, field dimensions, spatial degrees of freedom, accumulated days of practice*, and *playing time*) were modelled using smooth functions, whereas categorical predictors (*team balance, landscape representativity*, and *activity order after warming*) were included as parametric fixed effects. Given the repeated-measures structure of the dataset, with multiple observations nested within six GKs, GK identity was included as a random effect to account for within-player dependency and reduce pseudo-replication. Because PC was continuous and bounded between 0 and 1, models were specified using a Beta error distribution with a logit link function. PC values were transformed to the open interval (0,1) using the adjustment proposed by Smithson and Verkuilen [41] to avoid boundary values. Models were fitted using the *gam()* function from the *mgcv* package in R [42], with smoothing parameters estimated via Restricted Maximum Likelihood (REML). The basis dimension (*k*) for smooth terms was selected conservatively based on the number of unique observations available for each predictor (bounded by unique values − 1, with a maximum of 6). Model adequacy was evaluated using the diagnostic procedures implemented in *gam.check()*, including inspection of residual plots and assessment of the *k*-index to verify the adequacy of basis dimensions. For categorical predictors, overall effects were assessed using likelihood ratio tests (LRTs) comparing the full model with a reduced model including only the random effect. Differences between factor levels were interpreted from the parametric coefficients of the fitted models using the first level as the reference category. Statistical significance was set at *P* < 0.05.

## RESULTS

The PCs exhibited substantial variability across training tasks and GKs, ranging from 0.13 to 1 (Table 1). GAMMs revealed moderate non-linear effects of task constraints on GK performance. The degree of non-linearity varied across constraints, as indicated by effective degrees of freedom (edf) values ranging from 2.1 to 3.3 (Table 2). GK performance increased with the *number of players* involved in the task, but only up to a certain threshold, after which performance plateaued despite further increases in player numbers (edf = 2.2) (Figure 2A; Table 2). This model explained 28.9% of the deviance (χ^2^ = 29.8, *P* < 0.001). Similarly, *field dimensions* showed a com parable pattern, with performance increasing with larger playing areas before reaching a plateau (edf = 2.1). The model including *field dimensions* accounted for 20.8% of the deviance (χ^2^ = 18.7, *P* < 0.001) (Figure 2B; Table 2). *Spatial degrees of freedom* exhibited a stronger non-linear influence on PC, reflected by an edf of 2.6 and 27.8% of explained deviance (χ^2^ = 26.4, *P* < 0.001) (Figure 2C; Table 2). *Playing time* also displayed a pronounced non-linear relationship with PC (edf = 3.3), with the corresponding model explaining 30.0% of the deviance (χ^2^ = 21.6, *P* < 0.001) (Figure 2D; Table 2). In contrast, *accumulated days of practice* showed no relationship with PC (χ^2^ = 0.09, *P* = 0.93) (Table 2). GAMMs basis diagnostics indicated adequate smoothing parameter selection (*k*-index, *P* > 0.05) across models (Table 2). The random effect associated with GK identity was not statistically significant (*P* > 0.05) (Table 2).

**TABLE 1:** Values of Performance coefficients (PC) from the six study GKs across the training tasks (including GK specific tasks and tasks based on U16 category and U17 category). PC was calculated as the number of points achieved (pa) for each GK by the number or potential points (pp) that GKs would have achieved, i.e., based on the number of tries (expressed as a quotient, from 0 to 1). Where “nd” refers to no data available and N < 5 refers that PC were not considered due to number of actions were less than 5 for a GK and task (see methods for further details on PC calculation).

Training tasks	Type of task	U16			U17		

		PC GK 1 (pa / pp)	PC GK 2 (pa / pp)	PC GK 3 (pa / pp)	PC GK 4 (pa / pp)	PC GK 5 (pa / pp)	PC GK 6 (pa / pp)

1	GK specific	0.40	0.56	0.60	0.25	nd	nd
2	U16	0.67	0.83	N < 5	0.60	nd	nd
3	U16	0.71	N < 5	0.30	0.78	nd	nd
4	GK specific	0.13	0.65	0.15	0.50	0.73	nd
5	GK specific	0.45	0.57	0.58	0.58	nd	0.27
6	U16	0.50	0.40	0.70	nd	nd	0.59
7	GK specific	nd	0.67	0.38	0.54	0.33	0.62
8	GK specific	nd	0.57	0.27	0.75	0.13	0.60
9	GK specific	nd	0.24	nd	0.52	0.43	0.59
10	U17	nd	nd	nd	nd	0.57	0.46
11	GK specific	0.50	nd	nd	0.55	0.22	0.47
12	U17	nd	nd	nd	0.73	N < 5	0.43
13	U17	nd	nd	nd	nd	0.11	0.69
14	GK specific	0.45	0.54	0.57	0.50	0.45	0.48
15	GK specific	0.53	0.36	nd	0.45	0.34	0.35
16	U17	nd	nd	nd	0.52	0.53	0.38
17	U17	0.56	0.56	nd	nd	nd	nd
18	U16	1	0.47	nd	nd	nd	nd
19	U16	N < 5	0.64	nd	nd	nd	nd
20	U16	0.67	0.67	nd	nd	nd	nd
21	U16	0.72	0.69	nd	nd	nd	nd
22	GK specific	0.26	0.53	0.44	0.23	0.27	0.68
23	GK specific	0.11	0.25	0.22	0.20	nd	0.17
24	U17	nd	nd	nd	0.50	0.33	0.60
25	U17	nd	nd	nd	0.83	0.40	nd
26	U17	nd	nd	nd	0.55	0.44	nd
27	U16	0.44	0.71	0.73	nd	nd	nd
28	U17	nd	nd	nd	0.29	0.50	0.33
29	U16	0.54	0.83	0.67	nd	nd	nd
30	U16	0.55	0.69	0.67	nd	nd	nd
31	U17	nd	nd	nd	0.50	0.64	nd

**TABLE 2:** Results from Generalized Additive Mixed Models (GAMMs) examining the relationships between goalkeepers’ performance coefficient (PC; dependent variable) and continuous task constraints (*number of players, field dimensions, spatial degrees of freedom, accumulated days of practice*, and *playing time*). The table reports: (i) significance of smooth terms, including effective degrees of freedom (edf), chi-square statistic (χ^2^), P-value (*P*), and percentage of deviance explained; (ii) basis dimension diagnostics, including basis dimension (*k*), *k*-index, and associated *P*; and (iii) random effects for goalkeeper identity.

Variable	Approximate significant smooth terms				GAM basis checking Random effects (GK identity)					

	edf	χ^2^	*P*	Deviance Explained (%)	*k*	*k*-index	*P*	edf	χ^2^	*P*

*Number of players* (*n*)	2.2	29.8	< 0.001	28.9	6	1.1	0.88	1.9	3.2	0.14
*Field dimensions* (m^2^)	2.1	18.7	< 0.001	20.8	6	1.1	0.64	1.2	1.6	0.24
*Spatial degrees of freedom* (*n*/S_A_)	2.6	26.4	< 0.001	27.8	6	1.2	0.88	2.1	3.7	0.12
*Accumulated days of practice*	1.1	0.09	0.93	–	–	–	–	1.3	1.7	0.23
*Playing time* (s)	3.3	21.6	< 0.001	30.0	6	1.09	0.82	2.6	5.5	0.06

**FIG. 2:**
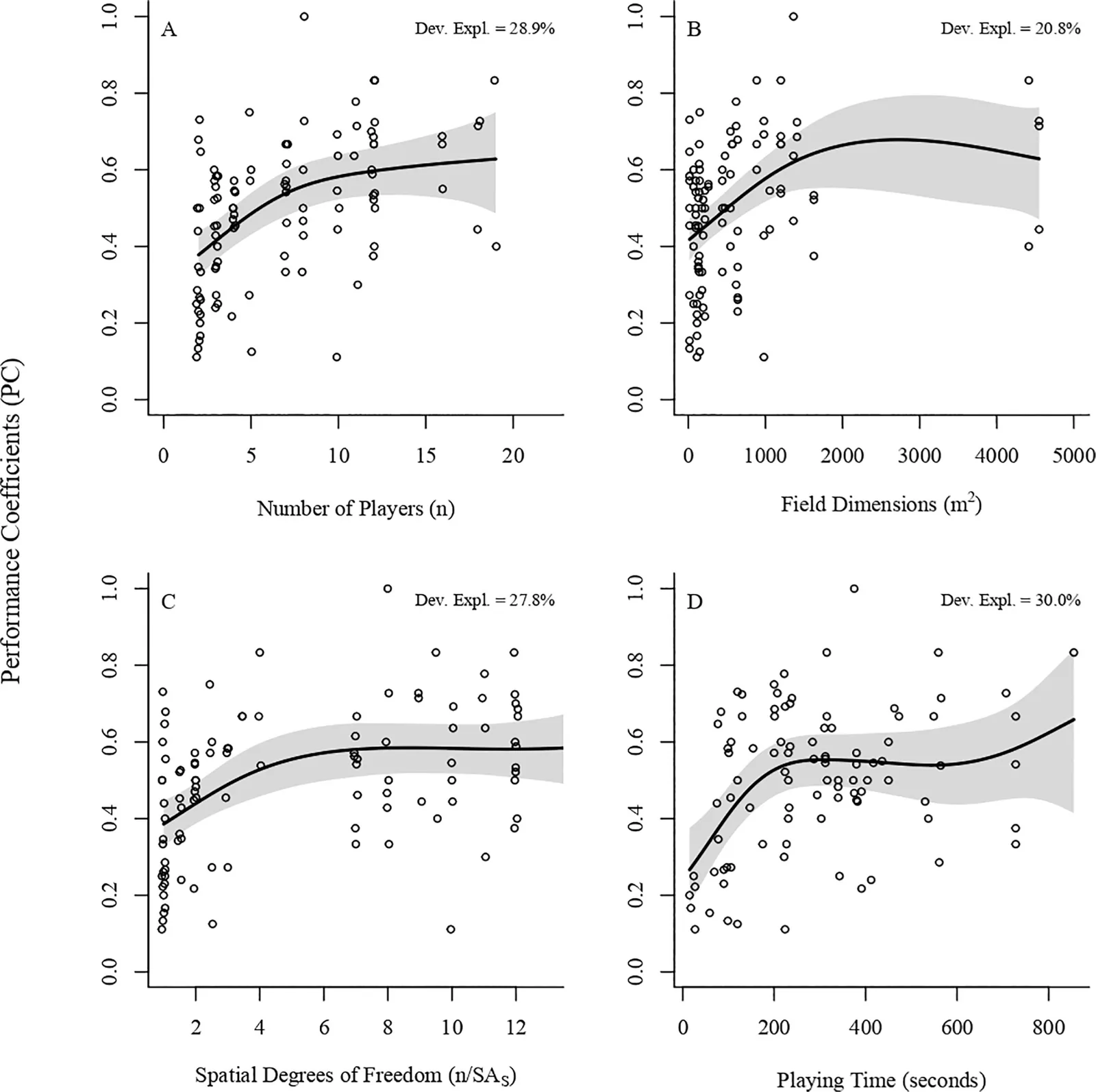
Non-linear relationships between GKs’ performance coefficients (PC) and continuous task constraints derived from univariate generalized additive mixed models (GAMMs). Panels show smooth effects for number of players (A), field dimensions (B), spatial degrees of freedom (C), and playing time (D). Solid lines represent estimated smooth functions, shaded areas indicate 95% confidence intervals, and points correspond to observed values. Deviance explained (%) is reported for each model.

Likelihood ratio tests (LRTs) indicated a significant overall effect of *landscape representativity* on PC (χ^2^ = 16.48, *P* = 0.003) (Table 3). Examination of the model coefficients showed that the 66.7% representativity level was associated with higher PC relative to the reference level (16.7%) (*z* = 2.42, *P* = 0.016), whereas other levels did not differ significantly (Table 3). In contrast, *team balance* did not show a significant overall effect (χ^2^ = 1.91, df = 1.32, *P* = 0.24), and *activity order after warming* showed only a non-significant trend (χ^2^ = 12.19, df = 6.76, *P* = 0.085) (Table 3).

**TABLE 3:** Global and specific effects of categorical task constraints on goalkeepers’ performance coefficient (PC) derived from univariate Generalized Additive Mixed Models (GAMMs). Upper panel presents likelihood ratio tests (LRTs; χ^2^) comparing full models with reduced models including only the random effect (GK identity). Lower panel shows Wald *z*-tests for individual contrasts. Estimates are reported on the logit scale (Beta distribution). Estimates represent contrasts relative to the reference level (first level of each categorical predictor). Significant effects (*P* < 0.05) are shown in bold.

**Global effects**	Variable	df	*χ* ^2^	*P*-value	
	*Team Balance*	1.32	1.91	0.24	
	*Landscape Representativity*	**4.40**	**16.48**	**0.003**	
	*Activity order after warming*	6.76	12.19	0.085	

**Specific Contrasts**	Variable	Levels	Estimate ± SE	*z*-value	*P*-value

	Team balance	1	0.30 ± 0.20	1.50	0.13
		3	0.10 ± 0.35	0.29	0.77
		5	0.52 ± 0.45	1.15	0.25

	Landscape Representativity	33.3%	0.11 ± 0.30	0.36	0.72
		50%	−0.024 ± 0.25	−0.10	0.92
		**66.7%**	**0.54 ± 0.22**	**2.42**	**0.016**
		83.3%	−0.17 ± 0.26	−0.65	0.52

	Activity order after warming	2	−0.13 ± 0.28	−0.46	0.64
		3	0.19 ± 0.26	0.73	0.47
		4	0.048 ± 0.26	0.18	0.86
		**5**	**0.63 ± 0.31**	**2.06**	**0.039**
		6	0.44 ± 0.38	1.16	0.25

## DISCUSSION

The results show positive relationships between GKs’ performance and several task constraints, such as the number of players involved, their spatial degrees of freedom and the dimensions of the playing area. This result supports our previous hypothesis that task constraints influence GKs’ performance. Specifically, the results indicate that training approaches that resemble “match-like” situations and promote dynamic player interactions may contribute to improve GK effectiveness, at least during training. Notably, the observed relationships between task constraints and performance show non-linear trends, as evidenced by the effective degrees of freedom derived from GAMMs, which range from 2.1 to 3.3. This suggests potential threshold-like effects in the relationship between task constraints and GKs’ performance, although this interpretation should be considered cautiously given the relatively small sample size. Overall, the results illustrate that GKs’ performance may be conceptualized as a complex interaction between the GKs and various properties of the training environment [2, 16, 33]. This supports a constraints-led approach in designing GK training programs [22, 37].

### The influence of task constraints on GKs’ performance

The results indicate a notable improvement in GKs’ performance in tasks characterized by a higher number of players interacting freely within the training area. This phenomenon implies that GKs might show heightened effectiveness in more complex and dynamic contexts, thereby fostering natural, “match-like” interactions among players. The increased frequency of player interactions is likely to enhance the awareness of various shared opportunities for action, which encompasses both collective opportunities among GKs, their teammates, and opposing players [12, 37, 43]. The ability to recognize and respond to these emergent opportunities might signifi-cantly affect GKs’ capabilities to perceive and anticipate the intentions of opponents, subsequently allowing for the execution of effective responses [44, 45]. For example, in a 3 v 2 shooting scenario, defenders create gaps that open and close, giving attackers transient chances for shots on goal. For GKs, a gap that offers an opportunity for a shot can also pose a challenge requiring a save. We suggest that a GK’s ability to respond and act on these dynamic gaps may enhance their performance. This aligns with the co-adaptation process that football players undergo during training [43, 46], where GKs demonstrate strong skills in perceiving and using complex perceptual-cognitive information [47].

The results show that constraints associated with training recurrence, specifically over the study period (i.e., 136 days), do not consistently correlate with performance outcomes. This observation underscores the argument that GKs’ skill acquisition cannot be solely attributed to the volume of practice over extended temporal scales, such as weeks to months [48, 49]. Conversely, at shorter temporal scales (i.e., minutes to hours), the amount of playing time exhibits a positive correlation with performance, signifying an apparent adaptive nature of GKs in response to immediate tasks. These contrasting results across different temporal scales illustrate that while skill acquisition tends to progress rapidly during the initial phases, it exhibits a decelerated trajectory over time [19, 20, 21]. Consequently, subsequent research that extends the duration of our study is warranted to further investigate the variability of goalkeeping skill acquisition across diverse temporal scales that reflect the nuances of training periodization.

In summary, the results manifest a significant influence of task constraints during GK training, as previously postulated in football [9, 14]. Based on the results, we suggest that GK training might be partially contextualized to reflect representative training scenarios. This interpretation suggests that skill acquisition and performance should not merely be perceived as a linear accumulation of training load. Instead, they should be also understood as a nuanced process of progressive attunement to relevant information within the training context, with training quality at the core of the design process [39, 46].

### Non-linear relationships between task constraints and GKs’ performance

The observed relationships between task constraints and performance exhibit non-linear trends, as highlighted by the effective degrees of freedom derived from GAMMs, ranging from 2.1 to 3.3. This reflects that the influence of task constraints on GKs’ performance may exhibit threshold-like effects, beyond which the effects of task constraints on performance might be less pronounced.

Specifically, GKs’ performance shows increasing-to-plateau tendencies with the *number of players* and their *spatial degrees of freedom*, as illustrated by values of edf ~ 2 from GAMMs. This can be interpreted as resembling “match-like” conditions during GK training necessitates a minimum number of player interactions, most likely to sufficiently generate representative information for the GKs. Accordingly, Bastias et al. [22] showed a foundational level of player interaction during GK training by engaging 4 to 5 GKs. These 4 to 5 GKs yielded performance outcomes comparable to those attained in more complex scenarios, including small-sided games (e.g., 5 *v* 5) and extensive training formats (e.g., 9 *v* 9) [22]. An apparent utility to consider player interaction during GK training is still speculative. However, the present study contributes to an evolving perspective that posits the inadequacy of “isolated” GK training, which is typically conducted with either a single GK or in tandem with merely one additional GK, and a coach [37].

In addition, the performance of GKs shows a moderate non-linear relationship with the *playing time*, as illustrated by values of edf = 3.3 from GAMMs. This complex model may reflect the processes of adaptation that GKs undergo during training, which encompass distinctly different developmental stages to the task at hand. Initially, there may be a marked increase in performance, characterized by rapid gains in performance during the early phase of training [19, 20], evolving into a more gradual enhancement later [21].

Collectively, the results underscore the significant influence of various task constraints on GKs’ performance, which may be largely non-linear. This suggests that the effects of task constraints on performance may vary across different ranges of the predictors, potentially indicating context-dependent responses. These results evidence that GK training is a complex process where performance is a context-dependent attribute of the performers. Therefore, the only application of linear training methodologies, predicated on the idealization of universal techniques or tactical patterns, may not be fully adequate to support GKs’ skill acquisition.

### Study limitations

The GAMMs in this study show a satisfactory fit to the data, with explained deviance between 20.8% and 30.0%. The *k*-indexes are close to 1 and have largely non-significant *P*-values (0.64 to 0.88), indicating optimal basis functions for modelling non-linearity. These results suggest that GAMMs are effective for analyzing the relationships between task constraints and GKs’ performance. However, the dataset included 102 observations derived from 22 training designs across six GKs, which may be susceptible to pseudo-replication. In the GAMMs, the random effect associated with GK identity was not statistically significant, suggesting limited between-player variability in performance responses to the task constraints. Another limitation of this study is that the sample primarily consists of elite youth female athletes, which restricts the generalizability of the findings. Future research should include a larger, more diverse sample to better understand the influence of task constraints on performance.

### Key take-home messages for practitioners

#### The non-linear framework of team-based training

Our study highlights the disproportionate relationship between task constraints and performance outcomes. For instance, increasing the number of players in a task may not enhance the information players can perceive and use effectively. Consequently, adjusting the number of players, under certain levels, may fall short of producing a commensurate enhancement in performance outputs; indeed, performance may even plateau or diminish contingent upon the contextual specifics of the training environment. Overall, the results indicate that task constraints have “boundaries of utility”, and their effectiveness may be highly context-dependent. Coaches therefore might systematically calibrate training designs and constraints to maximize player development based on specific contexts, such as squad composition, age group or season stage, ultimately enhancing training efficacy.

#### Representativeness on task design

The present study supports training to integrate representative tasks that closely mimic the dynamic and complex contexts of competitive match play. There exists controversy among coaches regarding the implementation of highly representative training methodologies, particularly concerning the potential decrease in the number of pure repetitions of a desired training episode (e.g., a shot at the edge of the penalty box). However, it is essential to recognize that players’ actions during matches are not solely governed by moments of ball contact. A considerable proportion of players’ performance is predicated on their ability to continuously adjust their positioning in the field, their own body set position, and effectively communicate with teammates [50], skills that may not be sufficiently cultivated through isolated and unopposed training tasks alone [51]. Consequently, it is advocated that a comprehensive training approach, harmonizing predictable exercises with representative and opposed tasks, may be optimal for player development.

#### Quality of practice over mere quantity and volume

Traditionally, skill development has been approached as a product of physiological fitness and repetitive practice drills, often conducted in isolation from competitive contexts [52]. However, our findings indicate that GKs’ performance is not exclusively related to the accumulated training, suggesting that factors beyond just training load are crucial for skill development. Supporting this notion, Bastias et al. [22] found that the accumulated days of practice only correlate with performance when additional constraints, such as task complexity, are taken into consideration. This implies that the sheer amount of practice does not necessarily lead to effective performance; rather, its effectiveness depends on the incorporation of qualitative factors [20, 21, 49]. Thus, training design may also emphasize the interactions between performers and their environments [48, 49], encouraging coaches to design rich learning environments that promote self-organizing tendencies, which emerge from the local interactions among players over time [25, 26].

## CONCLUSIONS

The present study supports the hypothesis that training designs resembling “match-like” conditions, and promoting dynamic player interactions, may contribute to improve GKs’ performance during training. The observed relationships between task constraints and performance are non-linear, suggesting that the effects of manipulating these constraints may be partially context-dependent. These findings provide preliminary support for the use of constraints-led principles in the design of GK tasks. In this context, coaches may act as mediators in the interactions between players and their training environments, thereby facilitating players’ abilities to attune to key environmental information, as previously suggested in other sports [37, 39, 43]. Future research involving larger and more diverse samples will help further clarify the role of task constraints in shaping goalkeeping performance across different training contexts.

## Data Availability

Data not shown and coding from this study is available under request.
